# Eccrine Porocarcinoma: A Case Report of a Rare and Aggressive Cutaneous Tumour

**DOI:** 10.7759/cureus.6244

**Published:** 2019-11-27

**Authors:** Soo Oh, Alireza Behzadnia, Jacquline Chan

**Affiliations:** 1 Surgery, Royal Stoke University Hospital, Stoke-on-Trent, GBR; 2 Histopathology, Royal Stoke University Hospital, Stoke-on-Trent, GBR; 3 Otolaryngology, Princess Royal Hospital, Telford, GBR

**Keywords:** porocarcinoma, eccrine, malignant neoplasm, adnexal tumour, rare tumor, adnexal mass, sweat gland neoplasm, cutaneous lesions, cutaneous neoplasm, poroma

## Abstract

Eccrine porocarcinoma (EPC) is an extremely rare, adnexal carcinoma that represents less than 0.01% of all cutaneous malignancies. An aggressive tumour with a high recurrence rate, it has a tendency to metastasise to regional lymph nodes. Once metastasis has occurred, mortality rate increases to 75%-80% and thus survival is dependent on adequate and timely resection of the lesion. EPCs are frequently missed as a differential diagnosis due to their rarity and non-specific appearance, which can lead to serious consequences for patients. Consequently, EPCs are an important diagnosis for clinicians to be aware of and consider when evaluating cutaneous lesions.

We present a case of EPC of the knee, which was initially misdiagnosed as a benign lesion on magnetic resonance imaging (MRI). We discuss the use of MRI in aiding assessment of EPCs.

## Introduction

Malignant cutaneous adnexal neoplasms are broadly divided into four groups of eccrine, apocrine, mixed, and un-classified tumours. Eccrine porocarcinoma (EPC), first described by Pinkus and Mehregan in 1963, is a rare type of adnexal carcinoma which accounts for less than 0.01% of all cutaneous malignancies [1-3]. It can present as a nodular, erosive plaque or polypoid growth that ulcerates. Porocarcinomas have a propensity to affect lower extremities in elderly patients. Characteristically, they are aggressive with a high rate of recurrence following excision and metastasis to regional lymph nodes. Metastasis has been shown to increase the mortality rate to 75%-80% [4] and thus survival rate is dependent on adequate and timely resection of the lesion. Diagnosis is based on histology from skin biopsy, however, initial assessment of large tumours may include imaging modalities such as magnetic resonance imaging (MRI). Although EPC is a histological diagnosis, it is important not to dismiss the role of imaging in guiding differential diagnosis. Currently, there is limited literature on imaging findings of EPC despite its wide range of mimics [5]. Here we present a case of EPC at the knee that was initially mistaken for a benign cyst on imaging and highlight the significance of education and awareness of EPC as a differential diagnosis of cutaneous neoplasms. We also discuss the use of MRI in aiding diagnosis of EPC. 

## Case presentation

**Figure 1:**
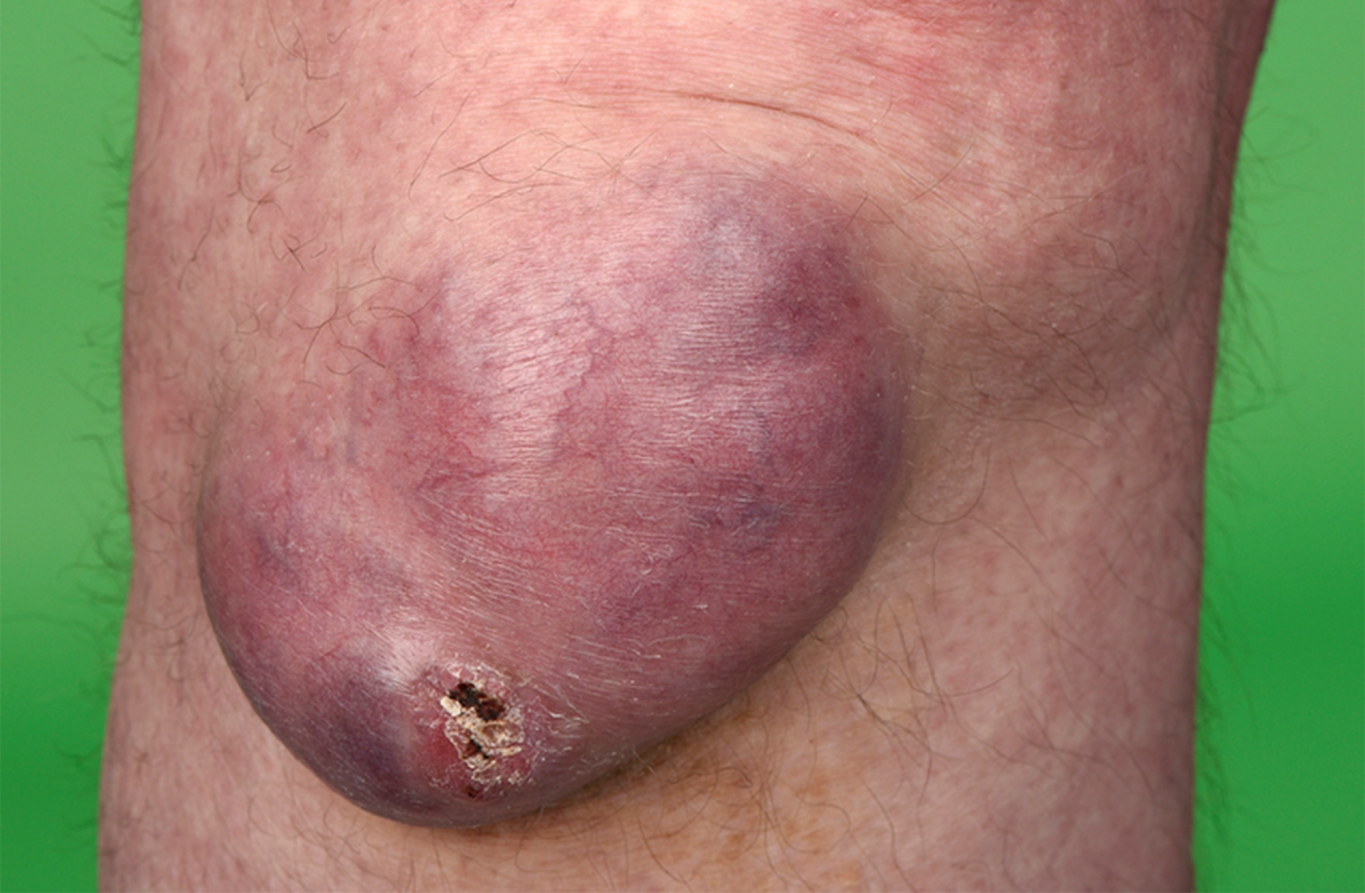
Clinical photography of the lesion on the right knee

**Figure 2:**
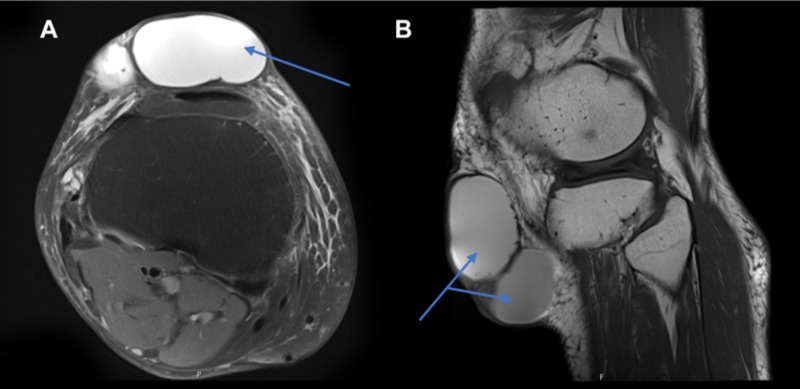
Magnetic resonance imaging (MRI) of the right knee with contrast showing loculated cystic mass A. Axial MRI of the right knee with gadolinium contrast showing a subcutaneous mass (see arrow) on the anterior aspect of the tibia and knee joint. It consists of a large loculated mass containing some debris inferiorly, lying superficial to and separate from the infrapatellar tendon. The mass has high signal on T1 with no enhancement around the cyst. B. Sagittal MRI of the right knee showing a subcutaneous mass (see arrows).

A 78-year-old male was referred to the dermatology outpatient department with a 10-year history of a slow-growing, large and fluctuant mass on his right knee. There was no associated discharge, pain or history of trauma. Clinical examination revealed a 7.5 cm x 7.5 cm x 3.5 cm purple lesion over the infrapatellar region with healthy overlying skin (Figure 1). There were no other lesions present elsewhere and no palpable regional lymphadenopathy. MRI with contrast of the knee identified a large loculated cyst lying anterior to the infra-patellar tendon with no communication with the knee joint. Additionally, a lateral meniscal cyst associated with an extensive meniscal tear was also identified (Figure 2). On consultation with the orthopaedic team, intra-articular communication was excluded and an opinion was sought from the plastic surgery team for excision of the cyst and reconstruction. Unfortunately, the patient became lost to follow up for two years before histological diagnosis. On re-presentation, the mass had increased in size and the patient was referred back to the plastic surgery department. This time, the lesion was excised and sent for histology.

Histopathological analysis showed a well-differentiated, partly cystic EPC. Depth was estimated to be 50 mm, Clarke level IV, stage pT3, with low mitotic activity at less than 14 per mm^2^. There was no evidence of vascular or perineural invasion. As the sample showed incomplete deep and peripheral margins, a wide local excision (WLE) with a larger 2 cm margin was undertaken. Histology of the re-excision sample showed clear histological margins with no residual tumour. A split-thickness skin graft was placed to expedite wound healing. The patient was regularly reviewed in the clinic until full wound healing was achieved and discharged to primary care with planned annual follow-up appointments for five years.

## Discussion

EPCs are rare malignant lesions most commonly affecting the lower extremities (35%), head and neck (24%), and upper extremities (14%). There is equal representation of gender and it is more commonly found in elderly patients (age range 42-90 years old) [6-7].

Surgical resection of the lesion is curative in 70%-80% of the cases, with a 20% risk of local recurrence and 20% risk of metastases to the regional lymph nodes. Metastatic disease has a poor prognosis with relative mortality of 50%-80% and a 10-year overall survival rate of 9% only [7]. Histopathological features also influence the prognosis of these lesions. Previous case reports have identified lymphovascular invasion, depth of invasion measured from the granular layer or ulcerated surface of the neoplasm to the deepest site of invasion, number of mitoses per 10 high-power fields (HPF) by ×40 objective and ×10 eye space and growth pattern of the neoplastic margin as being important prognostic factors [4]. It should be noted that these prognostic factors are based upon reviews of case reports in the literature and there is limited data available due to the rarity of EPCs. 

The pathogenesis of EPC remains unclear. Previously reported cases have been characterised by loss of heterozygosity of the TP53 gene [8-9]. Whole genomic profiling of a metastatic eccrine tumor undertaken by Thibodeau et al. identified somatic mutation of cyclin-dependent kinase inhibitor 2A/B gene, encoding for TP16 and TP14 as another possible carcinogenetic driver [10].

Traditionally, the pathogenesis of eccrine carcinomas was hypothesised to be due to the transformation of benign poromas to malignant porocarcinomas [5-6]. This was based on the typical clinical history of a slow-growing tumour (average 8.5 years) with a rapid change in size preceding the acute presentation [3,5,11]. Interestingly, eccrine poromas could share the same cell lineage as EPCs, as both have been found to be TP16 positive. However, TP16 is not a good marker for predicting malignant transformation of benign eccrine poromas [12]. 

Diagnostic work up of large cutaneous lesions often involves the use of MRI scanning for evaluation. However, accurate differentiation between benign and malignant lesions is highly challenging, especially in soft tissue lesions, due to their non-specific appearance [13]. In general, well-defined lesions that are smaller than 5 cm with homogenous signal intensity (SI), particularly on T2 weighted image (WI) MRI are benign in more than 90% of the cases [14]. Adnexal tumours are notoriously difficult to diagnose based on MRI and the data on their radiological features is scantily available. Chang et al. have proposed that “mushroom-like” exophytic skin appendages could be a suggestive feature of skin adnexal tumours, as those seen in mycosis fungoides [15]. However, no classical, defining feature has been determined, most likely due to the lack of well-documented MRI features of adnexal tumours and its rare incidence.

The varied and non-specific presentation of adnexal tumours makes correct diagnosis based on clinical assessment and imaging difficult. To confirm the diagnosis, a tissue biopsy is required for examination of histological features. In such instances, a WLE biopsy would achieve adequate marginal resection and with clear margins, both diagnostic and curative in most cases. Therefore WLE, rather than punch or shave biopsy, should be the method of biopsy in most cases.

## Conclusions

EPCs are a rare but important cause of cutaneous neoplasm. Patients can present through various referral pathways and may be assessed by numerous specialties prior to a diagnosis being made. Therefore, it is important for all clinicians to recognise EPC as a potential differential diagnosis for cutaneous lesions in order to prevent delays in treatment that could significantly affect patient outcomes. Lastly, further imaging data would be necessary in order to identify definitive MRI features for EPC.
